# Establishment of a risk stratification model based on the combination of post-treatment serum squamous cell carcinoma antigen levels and FIGO stage of cervical cancer for treatment and surveillance decision-making

**DOI:** 10.1007/s00432-022-04558-1

**Published:** 2023-01-09

**Authors:** Liu Shi, Yuxin Liu, Junyun Li, Jia Kou, Yi Ouyang, Foping Chen, Xiaodan Huang, Lanqing Huo, Lin Huang, Xinping Cao

**Affiliations:** Department of Radiation Oncology, State Key Laboratory of Oncology in South China, Collaborative Innovation Center for Cancer Medicine, Sun Yat-Sen University Cancer Center, No. 651 Dongfeng Eastern Road, Guangzhou, 510060 China

**Keywords:** Cervical squamous cell carcinoma, Squamous cell carcinoma antigen, FIGO stage, Risk stratification, Recursive partitioning analysis

## Abstract

**Objective:**

To develop a risk stratification model based on the International Federation of Gynecology and Obstetrics (FIGO) staging combined with squamous cell carcinoma antigen (SCC-Ag) for the classification of patients with cervical squamous cell carcinoma (CSCC) into different risk groups.

**Methods:**

We retrospectively reviewed the data of 664 women with stage IIA–IVB CSCC according to the 2018 FIGO staging system who received definitive radiotherapy from March 2013 to December 2017 at the department of radiation oncology of Sun Yat-sen University Cancer Center. Cutoff values for continuous variables were estimated using receiver operating characteristic curve analysis. Using recursive partitioning analysis (RPA) modeling, overall survival was predicted based on the prognostic factors determined via Cox regression analysis. The predictive performance of the RPA model was assessed using the consistency index (C-index). Intergroup survival differences were determined and compared using Kaplan–Meier analysis and the log-rank test.

**Results:**

Multivariate Cox regression analysis identified post-treatment SCC-Ag (< 1.35 ng/mL and > 1.35 ng/mL; hazard ratio (HR), 4.000; 95% confidence interval (CI), 2.911–5.496; *P* < 0.0001) and FIGO stage (II, III, and IV; HR, 2.582, 95% CI, 1.947–3.426; *P* < 0.0001) as the independent outcome predictors for overall survival. The RPA model based on the above prognostic factors divided the patients into high-, intermediate-, and low-risk groups. Significant differences in overall survival were observed among the three groups (5-year overall survival: low vs. intermediate vs. high, 91.3% vs. 76.7% vs. 29.5%, *P* < 0.0001). The predictive performance of the RPA model (C-index, 0.732; 95% CI, 0.701–0.763) was prominently superior to that of post-treatment SCC-Ag (C-index, 0.668; 95% CI, 0.635–0.702; *P* < 0.0001) and FIGO stage (C-index, 0.663; 95% CI, 0.631–0.695; *P* < 0.0001).

**Conclusions:**

The RPA model based on FIGO staging and post-treatment SCC-Ag can predict the overall survival of patients with CSCC, thereby providing a guide for the formulation of risk-adaptive treatment and individualized follow-up strategies.

**Supplementary Information:**

The online version contains supplementary material available at 10.1007/s00432-022-04558-1.

## Introduction

Globally, cervical cancer was the fourth leading cause of death among women in 2018 (Bray et al. 2018). The most common factors responsible for cervical cancer are stage, tumor size, lymph node status, depth of tumor invasion, and lymphovascular space invasion (Peters et al. 2000). Owing to the differences in the coverage of screening and preventive measures, there is a noticeable geographic variation in the incidence of cervical cancer. Cervical cancer is the third leading cause of cancer-associated death among women in mid-to-low income countries, which was seldom the case among women in high-income countries. Similarly, owing to discrepancies in proper management and follow-up, there is also a geographical variation in the disease's mortality rate. According to surveys, since the mid-1970s, the survival rate of the cervical cancer has been static, which primarily suggests the lack of significant treatment progress for patients with recurrent and metastatic diseases (Bray et al. 2018; Siegel et al. 2020; Torre et al. 2016). In a sense, early diagnosis of recurrence and timely salvage treatment may improve patients’ quality of life, prolong their life and improve the survival rate on a large scale. As a result, it is important to find an economical and sensitive way of monitoring this disease.

Approximately 75% of invasive cervical cancer cases are squamous cell carcinomas (Small et al. 2017). Emerging alongside the squamous formation of the uterine cervix, squamous cell carcinoma antigen (SCC-Ag) is elevated when the squamous epithelium of the cervix is neoplastically transformed (Maruo et al. 1998). In 28–88% of cervical squamous cell carcinoma (CSCC) cases, there is an increase in serum SCC-Ag levels, indicating that SCC-Ag could serve as an important biomarker for cervical cancer (Ohara et al. 2002; Dasari et al. 2015). Previous studies have reported that as a crucial indicator, SCC-Ag can help physicians in outcome prognosis, treatment decision-making, and recurrence detection (Liu and Shi 2019; Wang et al. 2019; Markovina et al. 2018).

Despite the extensive application of pre- and post-treatment serum SCC-Ag measurements for cervical cancer in clinical settings, there is no validated and consistent tool to help physicians judge whose disease is cured and who potentially faces possible recurrence. The official guidelines do not mention the likely scenarios for conducting adjuvant therapy. Therefore, most physicians use FIGO staging, imaging examination, SCC-Ag, tumor response rate, and cumulative experience for decision-making.

The present study developed a risk model for patients with cervical cancer who received radiotherapy and chemotherapy or radiotherapy alone to identify groups that may benefit from adjuvant therapy and establish more appropriate follow-up strategies.

## Materials and methods

In the present retrospective observational study, 664 women diagnosed with IIA–IVB CSCC who received definitive radiotherapy from March 2013 to December 2017 at the Department of Radiation Oncology of Sun Yat-sen University Cancer Center were enrolled.

﻿The inclusion criteria were as follows: (a) histologically diagnosed with stage IIA–IVB CSCC of the FIGO 2018 classification; (Bhatla et al. 2019) (b) thoracic and abdominal computed tomography (CT), pelvic magnetic resonance imaging (MRI), and positron emission tomography (PET)/CT assessment of lymph node status and tumor extension; and (c) primary management by a combination of definitive radiotherapy (with or without chemotherapy) and brachytherapy. The exclusion criteria were as follows: (a) patients with ﻿adenosquamous carcinoma, adenocarcinoma, small cell carcinoma, and other histological types of cervical cancer; (b) those without key data, such as serum SCC-Ag levels acquired before or after treatment and tumor size; and (c) those with a Karnofsky performance status of < 70.

All patients underwent external beam radiotherapy alone or in combination with chemotherapy and subsequent brachytherapy once a week (28 Gy delivered in four fractions or 30 Gy delivered in five fractions). The 5-week external beam radiotherapy was conducted by intensity-modulated radiation therapy (IMRT) or volumetric modulated arc therapy (VMAT), where the overall dosage was 45–60 Gy in 25 fractions. A radiation boost was delivered for those suspected of pelvic node involvement upon radioimaging, typically at a dose of10 Gy in five fractions.

A group of patients (407) received chemotherapy for different reasons and regimens. The neoadjuvant and adjuvant chemotherapy regimens included TP (docetaxel 75–100 mg/m^2^ d1 plus cisplatin 80–100 mg/m^2^ d1-2, q3w, 1–3 cycles) and FP (5-fluorouracil 300–500 mg/m^2^, d1-5 plus cisplatin 80–100 mg/m^2^ d1-2, q3w, 1–3 cycles). The concurrent chemotherapy regimens comprised cisplatin (50 mg/m^2^, d1, qw, 4–6 cycles or 80 mg/m^2^, d1-2, q3w, 2 cycles), nedaplatin (80 mg/m^2^, q3w, 2 cycles), TP (docetaxel 75 mg/m^2^ d1 plus cisplatin 80 mg/m^2^ d1-2, q3w, 2 cycles), and FP (5-fluorouracil 300 mg/m^2^, d1-5 plus cisplatin 80 mg/m^2^ d1-2, q3w, 2 cycles), with inconsistent doses and cycles.

Pre-treatment imaging examination (MRI, CT, or PET/CT) or CT simulation images before delineating the radiotherapy target volume revealed metastasis to the pelvic or para-aortic lymph node. Tumor size was defined as the longest diameter measured on MRI, CT, PET/CT, or physical examination before the initial treatment.

After completing the primary treatment, relapses in the vagina, cervix, uterus, and parametria were deemed as local recurrence; lesions in the lymph node (aortic or pelvic) were considered regional recurrence; and distant metastasis included recurrences in any distant organ or lymph node such as the lungs, brain, bone, and supraclavicular lymph nodes.

For every patient, pre-treatment levels of SCC-Ag were measured at diagnosis or before initial treatment. After finishing the curative treatment, post-treatment SCC-Ag was measured at approximately 4–8 weeks. Electrochemiluminescence immunoassay was performed for measuring serum SCC-Ag levels using a commercial ECLIA kit (Roche Diagnostics, Mannheim, Germany), where the upper normal limit was set at 1.5 ng/mL.

Overall survival, the primary study endpoint, was measured from treatment commencement to the final follow-up or the date of death from any cause. Progression-free survival, the secondary study endpoint, referred to a period from the initial therapy date to the date of local recurrence or metastasis.

Overall survival and progression-free survival were determined using the Kaplan–Meier approach combined with the log-rank test. Cutoff values for pre- and post-treatment SCC-Ag and tumor size were determined using the area under the ROC curve (AUC) analysis. Prognostic predictors were identified using Cox regression models by estimating hazard ratio (HR) and 95% confidence interval (CI). Age, FIGO overall stage, regional lymph node metastasis, tumor size, pre-treatment SCC-Ag, post-treatment SCC-Ag and treatment modality are included in the univariate analysis. On the other hand, the FIGO stage, post-treatment SCC-Ag, and recursive partitioning analysis (RPA) risk groups were differentiated by comparing Harrell's index of concordance (C-index). Incorporating FIGO stage and post-treatment SCC-Ag into a risk stratification model, the low-, intermediate-, and high-risk patients were classified using RPA.

Data were analyzed using R 4.1.2 plus SPSS 26.0 (SPSS Inc., Chicago, USA). All *P* values were double-tailed, and differences were considered significant when *P* < 0.05.

## Results

The detailed characteristics of the 664 patients with CSCC are presented in Table 1. The median duration of follow-up was 53.7 (IQR 40.9–68.7 months). Patients with FIGO stage III accounted for 63.9% of the population. The 5-year overall survival rate and corresponding 95% CI was 70.2% (66.5–73.9%) and that of stage II, III, and IV was 88.5% (83.2–93.8%), 69.5% (64.8–74.2%), and 22.4% (9.5–35.3%), respectively. Overall, 184 (37.7%) patients died during the follow-up period owing to any causes. In 190 (28.6%) relapsed patients, 35 (5.3%) had local recurrence, 40 (6.0%) had regional relapse, and 115 (17.3%) had distant metastasis.

**Table 1:** Patients characteristics

Variables (*n* = 664)	
Age (median [IQR])	56[50, 63]
FIGO Stage (%)	
II	177 (26.7)
IIA1	32(4.8)
IIA2	14(2.2)
IIB	131(19.7)
III	424(63.9)
IIIA	25(3.8)
IIIB	80(12.0)
IIIC1r	278(41.9)
IIIC2r	41(6.2)
IV	63(9.5)
IVA	23(3.5)
IVB	40(6.0)
Regional lymph node involvement (%)	
Negative	300 (45.2)
Positive	364 (54.8)
Tumor size (cm)(median [IQR])	4.8 [3.9, 5.6]
< 4.05(%)	223(33.6)
> 4.05(%)	441(66.4)
Pre-treatment SCC-Ag (ng/mL) (median [IQR])	7.9 [2.6, 25.0]
< 19.25(%)	468(70.5)
> 19.25(%)	196(29.5)
Post-treatment SCC-Ag (ng/mL) (median [IQR])	0.8 [0.5, 1.2]
< 1.35(%)	528 (79.5)
> 1.35(%)	136 (20.5)
Status (%)	
Alive	480 (72.3)
Died for any cause	184 (27.7)
Follow-up time (months) (median [IQR])	53.7 [40.9, 68.7]
Relapse (%)	190(28.6)
Local	35(5.3)
Regional	40(6.0)
Distant	115(17.3)
Treatment modality (%)	
RT	257(38.7)
RT + NACT/ACT	119(17.9)
CCRT	145(21.8)
CCRT + NACT/ACT	143(21.5)

FIGO, International Federation of Gynecology and Obstetrics; SCC-Ag, squamous cell carcinoma antigen; RT, radiotherapy; NACT, neoadjuvant therapy; ACT, adjuvant therapy; CCRT, concurrent chemoradiotherapy.

**Fig. 1:**
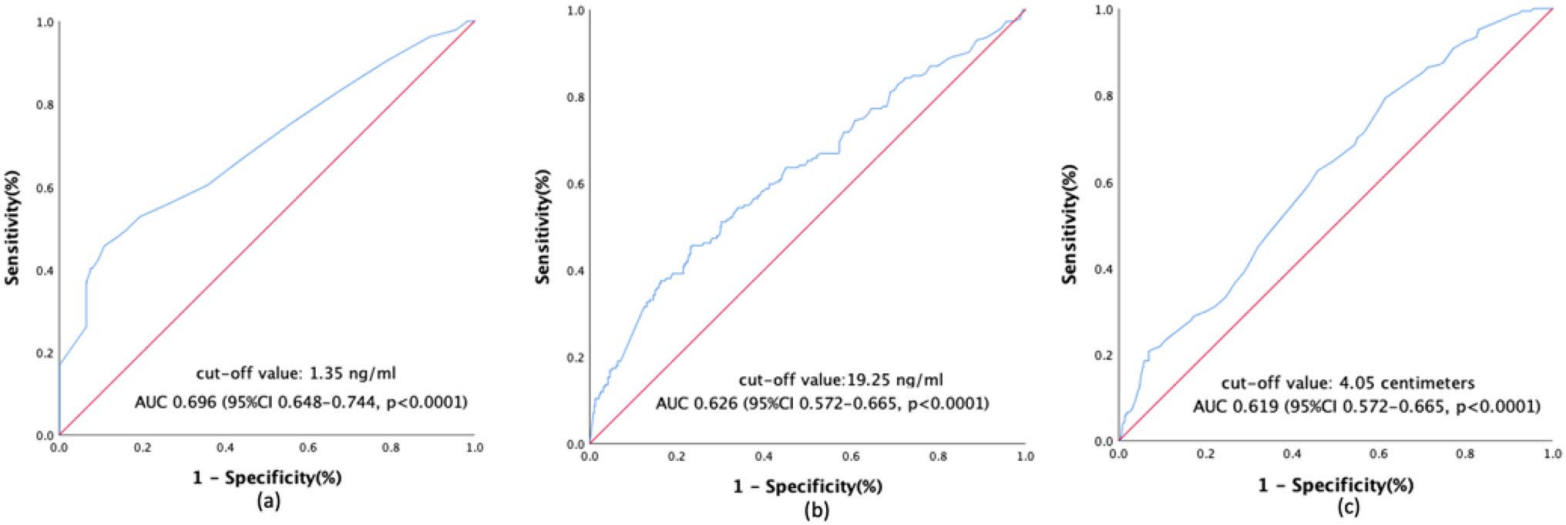
Receiver operating curves for post-treatment (**a**) and pre-treatment (**b**) SCC-Ag level and tumor size (**c**) in predicting overall survival in CSCC patients

Before the treatment, the median tumor size was 4.8 (IQR 3.9–5.6 cm). To differentiate the risks, the cutoff values for primary tumor size of 4.05 cm (sensibility, 79.3% and specificity, 38.5%) were derived using the ROC curve, in which the AUC was 0.619 (95% CI, 0.572–0.665; *P* < 0.0001) (Fig. 1c).

The median pre-treatment SCC-Ag level was 7.9 (IQR 2.6–25.0 ng/mL), whereas the median post-treatment SCC-Ag level was 0.8 (IQR 0.5–1.2 ng/mL). A prominent increase in post-treatment SCC-Ag level was observed with FIGO stage progression (0.75 ng/mL at stage II, 0.9 ng/mL at stage III, and 5.6 ng/mL at stage IV; *P* < 0.0001); however, the pre-treatment SCC-Ag level did not increase with the stages (31.7 ng/mL at stage II, 9.8 ng/mL at stage III, and 16.35 ng/mL at stage IV; *P* < 0.0001). Similarly, cutoff determination for pre- and post-treatment SCC-Ag was accomplished using the ROC curve for predicting survival so as to select the optimal SCC-Ag cutoff value for risk discrimination. The AUC was 0.626 (95% CI, 0.572–0.665; *P* < 0.0001) and 0.696 (95% CI, 0.648–0.744; *P* < 0.0001) for pre- and post-treatment SCC-Ag. Accordingly, the cut-off levels were 19.25 ng/mL (sensibility, 45.7% and specificity, 76.7%) and 1.35 ng/mL (sensibility, 45.7% and specificity, 89.2%), respectively (Fig. 1a and b).

**Fig. 2:**
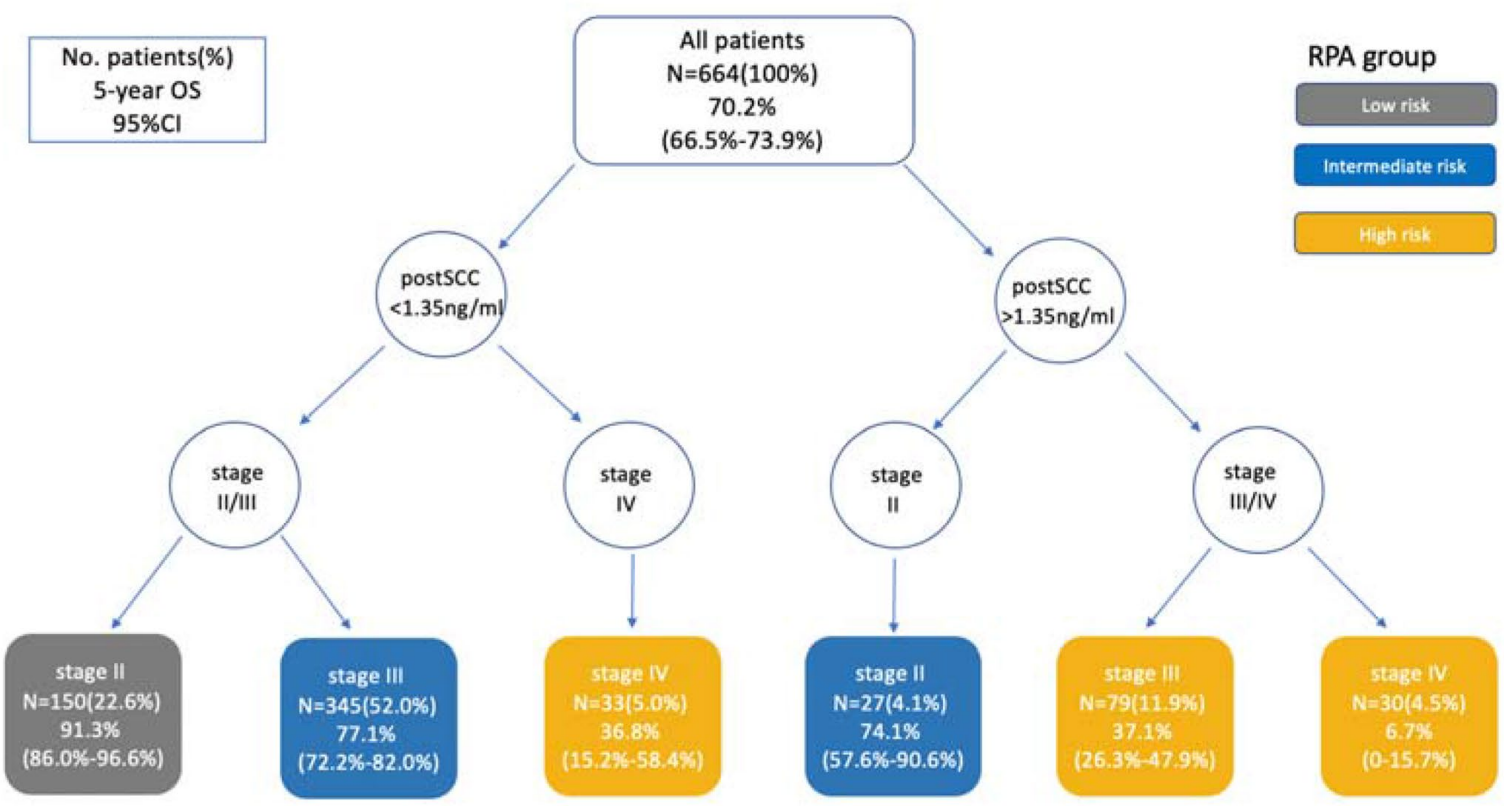
Risk classification tree by RPA. RPA, recursive partitioning analysis; OS, overall survival; CI, confidence interval

In univariate Cox regression analysis, post-treatment SCC-Ag (HR, 5.185; 95% CI, 3.865–6.957; *P* < 0.0001) was the foremost prognostic predictor for overall survival among the CSCC population, followed by FIGO stage (HR, 3.656; 95% CI, 2.805–4.765; *P* < 0.0001), pre-treatment SCC-Ag (HR, 2.294; 95% CI, 1.716–3.066; *P* < 0.0001), regional metastasis to the lymph nodes (HR, 2.162; 95% CI, 1.577–2.965; *P* < 0.0001), and tumor size (HR, 2.122; 95% CI, 1.485–3.033; *P* < 0.0001) (Supplementary Table S1). In multivariate Cox regression analysis, FIGO stage (HR, 2.582; 95% CI, 1.947–3.426; *P* < 0.0001) and post-treatment SCC-Ag (HR, 4.000; 95% CI, 2.911–5.496; *P* < 0.0001) (Supplementary Table S1), which were included in the RPA model, were the most significant predictors (Fig. 2).

**Fig. 3:**
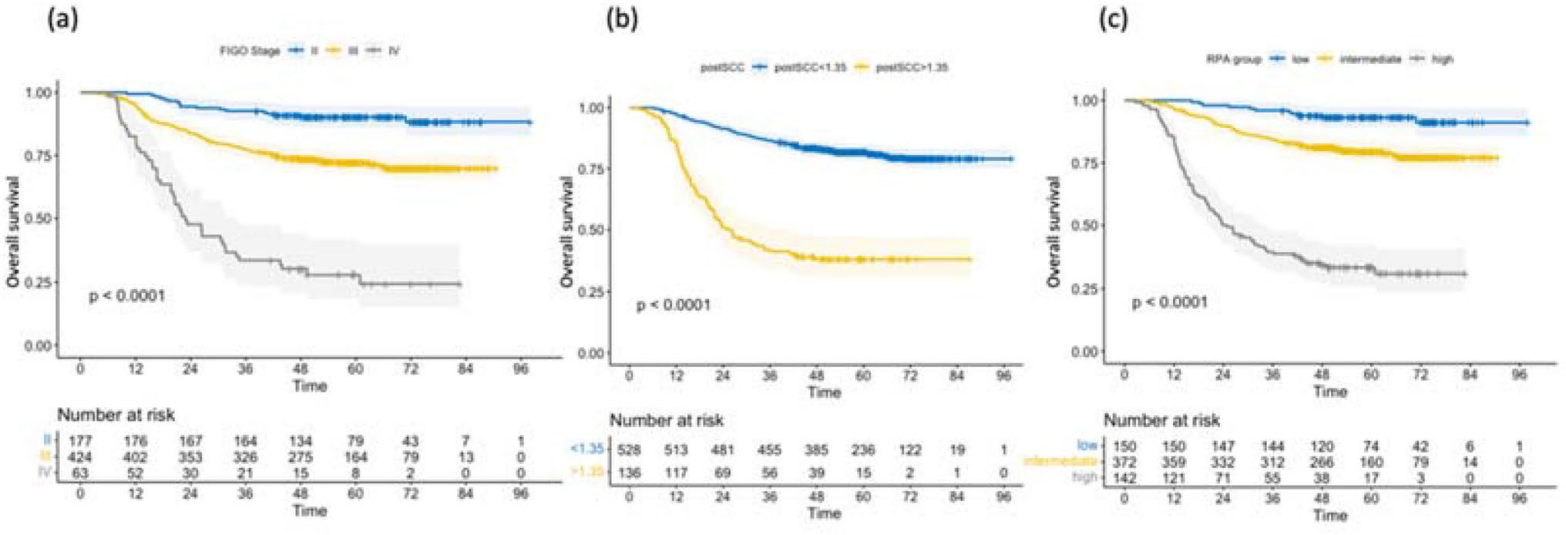
The Kaplan–Meier curves of overall survival by **a** FIGO stage, **b** post-SCC, **c** RPA group. FIGO, International Federation of Gynecology and Obstetrics; post-SCC, post-treatment squamous cell carcinoma antigen; RPA, recursive partitioning analysis

The RPA model stratified the patients into three different groups: low-, intermediate-, and high-risk groups. In terms of survival rates, the 5-year overall survival rate of these three groups was 91.3% (95% CI, 86.0–96.6%), 76.7% (95% CI, 71.8–81.6%), and 29.5% (95% CI, 20.3–38.7%), respectively. Further, the 5-year overall survival rate of post-treatment SCC-Ag < 1.35 ng/mL and > 1.35 ng/mL was 78.7% (95% CI, 74.8–82.6%) and 37.7% (95% CI, 29.5–45.9%) and that of FIGO stage II, III, and IV was 88.5% (95% CI, 83.2–93.8%), 88.5% (95% CI, 83.2–93.8%), and 22.4% (95% CI, 9.5–35.3%), respectively (Fig. 3 and Supplementary Table S2).

There was noticeable insulation of survival rates using the Kaplan–Meier approach in each prognostic group. The RPA groups had a more segregated hazard gap than either stage or post-treatment SCC-Ag alone (low-risk: reference; intermediate-risk: 3.107, 95% CI, 1.652–5.841; and high-risk: 16.064, 95% CI, 8.585–30.057) (Fig. 3 and Supplementary Table S2).

The C-index for overall survival was 0.732 (95% CI, 0.701–0.763) in the RPA risk group, which was significantly increased from that for post-treatment SCC-Ag (0.668; 95% CI, 0.635–0.702; *P* < 0.0001) and FIGO stage (0.663; 95% CI, 0.631–0.695; *P* < 0.0001) alone (Table 2).

**Table 2:** Comparison of the c-index of the RPA groups, post-treatment SCC-Ag, and FIGO stage

Risk group	C-index (95% CI)	*P* value
RPA risk group	0.732 (0.701–0.763)	Ref
Post-treatment SCC-Ag	0.668 (0.635–0.702)	< 0.0001
FIGO stage	0.663 (0.631–0.695)	< 0.0001

FIGO, International Federation of Gynecology and Obstetrics; RPA, recursive-partitioning analysis.

Among the 664 patients, 257 (38.7%) completed definitive radiotherapy without concomitant chemotherapy, while the remaining 407 (61.3%) patients received different chemotherapy regimens before, during, or after radiotherapy. Among the patients who received chemotherapy, 145 (21.8%) received concurrent chemoradiotherapy, 134 (21.5%) received concurrent chemoradiotherapy and neoadjuvant chemotherapy/adjuvant chemotherapy, and 119 (17.9%) received radiotherapy and neoadjuvant chemotherapy/adjuvant chemotherapy (Table 1).

**Fig. 4:**
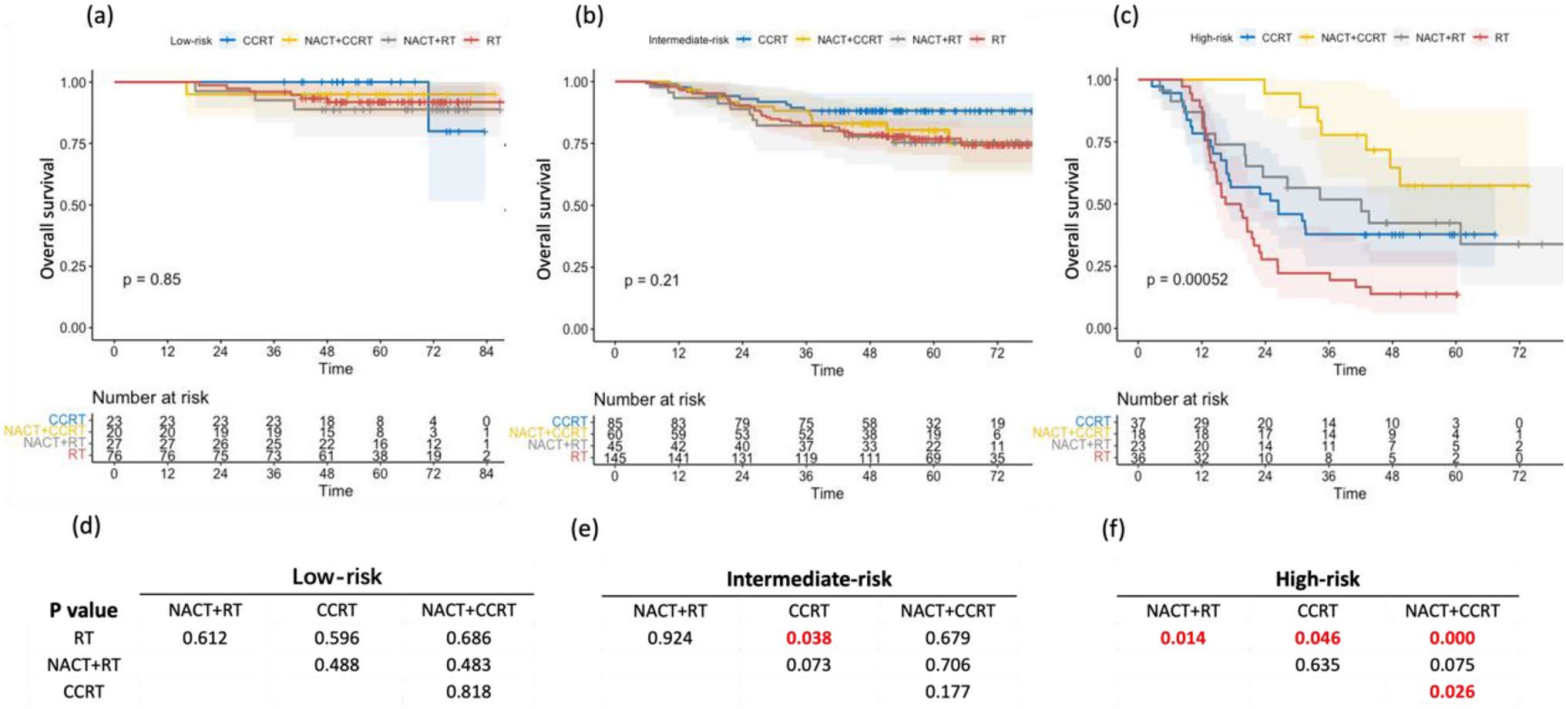
The Kaplan–Meier curves and the paired comparisons of overall survival by different treatment protocols in RPA low-risk (**a**,** d**), intermediate-risk (**b**, **e**), and high-risk (**c**, **f**) group. RPA, recursive partitioning analysis; RT, radiotherapy; NACT, Neoadjuvant therapy; CCRT, concurrent chemoradiotherapy

In the RPA risk groups, the low-risk group had a higher number of patients (76/150, 50.7%) who received only definitive radiotherapy. In contrast, compared to the other two groups, a considerable number of patients in the high-risk group (37/142, 26.1%) received concurrent chemoradiotherapy, which was almost similar to the number of patients receiving concurrent chemoradiotherapy and neoadjuvant chemotherapy/adjuvant chemotherapy (39/142, 27.5%) (Supplementary Table S3). In terms of the effect of therapeutic modality on overall survival, the risk groups had radically different performances. Insignificant differences were observed among the treatment modes for the low-risk group (Fig. 4a and d and Supplementary Figure S1a). However, the patients in the intermediate-risk group receiving simultaneous chemoradiotherapy exhibited prominently superior overall survival compared with those receiving radiotherapy (*P* = 0.038) (Fig. 4b and e). In contrast, the prognosis of concurrent chemoradiotherapy followed by neoadjuvant chemotherapy preceded that of other chemotherapy regimens in the high-risk group and was particularly better than that of radiotherapy (*P* = 0.000) and concurrent chemoradiotherapy (*P* = 0.026) (Fig. 4c and f).

## Discussion

Our study identified that post-treatment SCC-Ag has a potentially more significant relationship with overall survival than pre-treatment SCC-Ag, whose high expression signified worse survival, particularly when the value was higher than 1.35 ng/mL. We performed multivariate Cox regression analysis to identify the most influential factors—FIGO 2018 stage and post-treatment SCC-Ag, making further efforts to develop an RPA risk model by combining FIGO stage and post-treatment SCC-Ag to stratify patients into the high-, intermediate-, and low-risk groups. The model had preferable prediction and discrimination efficiency for overall survival compared with either parameter.

Previous studies have verified the close relationship between SCC-Ag and the outcomes of patients with CSCC. ﻿A meta-analysis selected 17 articles and concluded that high SCC-Ag expression is linked to poor prognosis of patients with CSCC (Liu and Shi 2019). However, it did not discuss the SCC-Ag levels at different time points. Some studies have reported the close relationship between pre-treatment SCC-Ag and the risk of death and recurrence (Guo et al. 2020; Choi et al. 2018). However, in this study, probably because of differences in measuring timing points or restriction of sample size, pre-treatment SCC-Ag was not considered an outcome predictor via Cox regression analysis. Some other studies have reported the prognostic role of post-treatment SCC-Ag (Wang et al. 2019; Benito et al. 2021; Salvatici et al. 2016). Benito et al. (2021) detailed the prophetic role of post-treatment SCC-Ag. They analyzed 447 patients with cervical cancer with IB2-IVA and identified that post-treatment SCC-Ag ≥ 1.2 ng/mL is an independent risk predictor for patients with cervical cancer with local advancement through multivariate Cox regression analysis (HR, 1.95; 95% CI, 1.11–3.44; *P* = 0.02); this indicates that those with post-treatment SCC-Ag of ≥ 1.2 ng/mL exhibit poor overall survival, similar to our findings.

According to the latest version (2022) of the National Comprehensive Cancer Network guidelines for cervical cancer (Abu-Rustum et al. 2022), the most recommended primary treatment for surgically inappropriate patients with cervical cancer with local advancement is individualized external beam radiotherapy and simultaneous Pt-containing chemotherapy with subsequent brachytherapy. However, the administration of pre-radiotherapy neoadjuvant chemotherapy or/and post-radiotherapy adjuvant chemotherapy remains controversial. For patients with distant metastasis, that is, FIGO stage IVB, most studies have reported that systemic treatment comprising Pt-containing chemotherapy, immunotherapy, and targeted therapy should be considered the mainstream treatment and that individualized radiotherapy should be selected as a complementary treatment (Abu-Rustum et al. 2022; Cohen et al. 2019; Dyer et al. 2019; Marquina et al. 2018). da Costa et al. (2019) conducted a phase II clinical trial and concluded that the survival benefit of concurrent chemoradiotherapy and neoadjuvant chemotherapy (gemcitabine and cisplatin) had worse efficacy than chemoradiotherapy alone for managing patients with cervical cancer with local advancement and that toxicities were more common. However, this study lacked subgroup analysis based on tumor size, lymph node diameter, or some other prognostic factors. Yavas et al. (2019) and Yuan et al. (2022) recommend adjuvant chemotherapy after simultaneous chemoradiotherapy for patients with locally advanced cervical cancer (LACC). The former claimed that adjuvant chemotherapy could improve both disease-free survival (DFS) and overall survival of patients with LACC and the latter showed that for patients with pelvic lymph node-positive CSCC, 3-year DFS can be increased without increasing side effects.

However, Kou et al. (2022) disagreed with this view because they found that adjuvant chemotherapy was not beneficial for the prognosis of patients and possibly led to a higher risk of side effects. A prospective study (NCT02036164) (Tangjitgamol et al. 2019; Tovanabutra et al. 2021) also indicated that adjuvant chemotherapy in combination with concurrent chemoradiotherapy could not improve the response rate or survival benefits of patients with LACC without para-aortic lymph node enlargement. The results of ongoing or forthcoming large-scale clinical trials are warranted (i.e., ACTRN12610000732088) to verify these views.

The present study explored the effect of each treatment regimen on each RPA group. We conclude that the patients in the low-risk group can choose any type of treatment if the adverse effects are not taken into consideration. Therefore, radiotherapy alone is enough for these patients, which could spare the toxicity of chemotherapy without worrying about its effects on prognosis and can help clinical physicians make treatment decisions. For the intermediate- and high-risk groups, adjuvant chemotherapy did not have an effect on survival rates at a statistical level. However, because one of the prognostic factors is post-treatment SCC-Ag, this prediction model cannot provide suggestions for the timing of receiving neoadjuvant chemotherapy or concurrent chemotherapy.

Furthermore, a previous study has reported that enhancement in the recurrence detection sensitivity (49.1% vs. 88.7%, *P* < 0.001) was attained by introducing SCC-Ag determination into the guideline-recommended protocol of follow-up (Oh et al. 2016). Compared with conventional imaging examination, physical examination, and pathological biopsy, SCC-Ag level was more accessible and affordable during the long-term follow-up period. For the patients in the low-risk group, the frequency of follow-up can suitably reduce while it can increase for those in the high-risk group, which can rationally allocate and considerably save scarce imaging resources and relieve patients' burden, particularly in developing countries.

To the best of our knowledge, the present study is the first and largest of its kind that combines tumor markers and stages to develop a risk prediction and stratification tool for treatment and follow-up decisions for patients with CSCC. Nevertheless, it has some limitations. First, some parameters are inevitable, such as sample selection bias, variety of treatment modalities, and sorting of crucial data. Second, internal and external data validation was not conducted owing to the limited sample size and source. Lastly, the post-treatment SCC-Ag cutoff value remains to be affirmed in a future study.

The RPA model constructed in the present study suggests that adjuvant chemotherapy is unnecessary for patients in the low-risk group. Although it could not assist physicians in developing treatment regimens for high- and intermediate-risk groups before therapy, it inspires us to conduct future investigations to identify more advantageous treatment methods in the corresponding subgroup, which is beneficial for designing future clinical trials.

Furthermore, by relying on the stratification ability of the RPA model and the role of serum SCC-Ag in follow-up, doctors can establish different surveillance strategies for diverse risk groups. For example, high-risk patients should be paid more attention to and monitored more frequently and strictly, whereas low-risk patients could decrease their frequency of reexamination, which would be a more rational and cost-effective approach.

In conclusion, post-treatment SCC-Ag and FIGO stage are the most meaningful predictive factors for survival. We built a simple and convenient model by integrating the post-treatment SCC-Ag and FIGO stage to efficiently stratify patients with CSCC. This model can assist physicians in preparing optimal treatment and surveillance schedules after primary treatment and will make it easier to promote and be widely used in clinical settings. In the future, we will conduct further studies aiming at the weaknesses of the present study.

## Supplementary Information

Below is the link to the electronic supplementary material.

Supplementary file1 (PDF 278 kb)

## Data Availability

The datasets generated during and/or analyzed during the current study are available from the corresponding author on reasonable request.
