# Supplementary material for: Establishment of a risk stratification model based on the combination of post-treatment serum squamous cell carcinoma antigen levels and FIGO stage of cervical cancer for treatment and surveillance decision-making
Source: J Cancer Res Clin Oncol. 2023 Jan 9;149(9):5999–6007. doi: 10.1007/s00432-022-04558-1 (PMC10356645; doi:10.1007/s00432-022-04558-1)
Supplement: Supplementary file 1 — Supplementary file1 (PDF 278 kb) [file 432_2022_4558_MOESM1_ESM.pdf]

Title of the article: Establishment of a risk stratification model based on the combination of post-treatment serum squamous cell carcinoma antigen levels and FIGO stage of cervical cancer for treatment and surveillance decision-making

Journal name: Journal of Cancer Research and Clinical Oncology

Corresponding author:

*Xinping Cao, Postal address: Department of Radiation Oncology, State Key Laboratory of Oncology in South China, Collaborative Innovation Center for Cancer Medicine, Sun Yat-Sen University Cancer Center, No. 651 Dongfeng Eastern Road, Guangzhou, 510060, China; Email address: caoxp@sysucc.org.cn

All author names: Liu Shi[1#], Yuxin Liu[1#], Junyun Li[1#], Jia Kou[1], Yi Ouyang[1], Foping Chen[1], Xiaodan Huang[1], Lanqing Huo[1], Lin Huang[1], Xinping Cao[1*].

Affiliations: [1]Department of Radiation Oncology, State Key Laboratory of Oncology in South China, Collaborative Innovation Center for Cancer Medicine, Sun Yat-Sen University Cancer Center.

Table S1 Univariate and multivariate analyses of the prognostic factors for overall survival (n = 664).

**Factors**	**Univariate analysis**		**Multivariate analysis**	
	HR (95% CI)	P value	HR (95% CI)	P value
**Age**	1.006 (0.991–1.022)	0.42	-	-
**FIGO Overall stage** (II, III, IVAB)	3.656 (2.805–4.765)	<0.0001	2.582 (1.947–3.426)	<0.0001
**Regional lymph node metastasis** (Yes, No)	2.162 (1.577–2.965)	<0.0001	1.237(0.872–1.754)	0.2336
**Tumor size** (<4.05cm, >4.05cm)	2.122 (1.485–3.033)	<0.0001	1.336(0.918–1.944)	0.131
**Pre-treatment SCC-Ag** (<19.25ng/mL, >19.25ng/mL)	2.294 (1.716–3.066)	<0.0001	1.273(0.928–1.748)	0.135
**Post-treatment SCC-Ag** (<1.35 ng/mL, >1.35 ng/mL)	5.185(3.865–6.957)	<0.0001	4.000(2.911–5.496)	<0.0001
**Treatment modality** (RT, RT+NACT/ACT, CCRT, CCRT+NACT/ACT)	1.013(0.8967–1.143)	0.841	-	-

Abbreviations： CI: confidence interval, FIGO: International Federation of Gynecology and Obstetrics, SCC-Ag: squamous cell carcinoma antigen, RT: radiotherapy, NACT: neoadjuvant therapy, ACT: adjuvant therapy, CCRT: concurrent chemoradiotherapy

Table S2 Overall survival and progression-free survival of the prognostic groups stratified based on FIGO stage, post-treatment SCC-Ag, and RPA risk groups

prognostic groups	Patient cohort(n = 664)						
	no. (%)	5-year OS (95%CI)	HR (95%CI)	P value	5-year PFS (95%CI)	HR (95%CI)	P value
**FIGO Overall stage**							
stage II	177 (26.7)	88.5% (83.2%-93.8%)	Reference		83.4% (77.7%-89.1%)		
stage III	424 (63.9)	69.5% (64.8%-74.2%)	3.125 (1.904–5.128)	<0.0001	69.2% (64.5%-73.9%)	2.085 (1.384–3.142)	0.0004
stage IV	63 (9.5)	22.4% (9.5%-35.3%)	12.393 (7.168–21.426)	<0.0001	34.1% (20.8%-47.4%)	6.229 (3.799–10.211)	<0.0001
**Post-treatment SCC-Ag**							
<1.35ng/mL	528 (79.5)	78.7% (74.8%-82.6%)	Reference		74.2% (70.3%-78.1%)	Reference	
>1.35ng/mL	136 (20.5)	37.7% (29.5%-45.9%)	5.185 (3.865–6.957)	<0.0001	53.4% (44.4%-62.4%)	2.575 (1.884–3.519)	<0.0001
**RPA risk group**							
low risk	150 (22.6)	91.3% (86.0%-96.6%)	Reference		84.0% (77.9%-90.1%)	Reference	
intermediate risk	372 (56.0)	76.7% (71.8%-81.6%)	3.107 (1.652–5.841)	0.0004	72.7% (67.8%-77.6%)	1.875 (1.191–2.952)	0.0067
high risk	142 (21.4)	29.5% (20.3%-38.7%)	16.064 (8.585–30.057)	<0.0001	46.4% (37.2%-55.6%)	5.282 (3.281–8.503)	<0.0001

Abbreviations: FIGO: International Federation of Gynecology and Obstetrics, SCC-Ag: squamous cell carcinoma antigen, HR: Hazard Ratio, CI: confidence interval

Table S3: Proportion of patients receiving different treatment modalities in the RPA groups.

**Treatment modality**<br>**No. of patients (%)**	**Low-risk group**<br>**(n = 150)**	**Intermediate-risk group**<br>**(n = 372)**	**High-risk group**<br>**(n = 142)**
**RT**	**76(50.7)**	**145(39.0)**	**36(25.4)**
**RT+NACT/ACT**	**29(19.3)**	**60(16.1)**	**30(21.1)**
NACT+RT	27(18.0)	45(12.1)	23(16.2)
NACT+RT+ACT	1(0.7)	12(3.2)	5(3.5)
RT+ACT	1(0.7)	3(0.8)	2(1.4)
**CCRT**	**23(15.3)**	**85(22.8)**	**37(26.1)**
**CCRT+NACT/ACT**	**22(14.7)**	**82(22.0)**	**39(27.5)**
NACT+CCRT	20(13.3)	60(16.1)	18(12.7)
NACT+CCRT+ACT	1(0.7)	16(4.3)	13(9.2)
CCRT+ACT	1(0.7)	6(1.6)	8(5.6)

Abbreviations: RPA: recursive partitioning analysis, RT: radiotherapy, NACT: neoadjuvant therapy, ACT: adjuvant therapy, CCRT: concurrent chemoradiotherapy

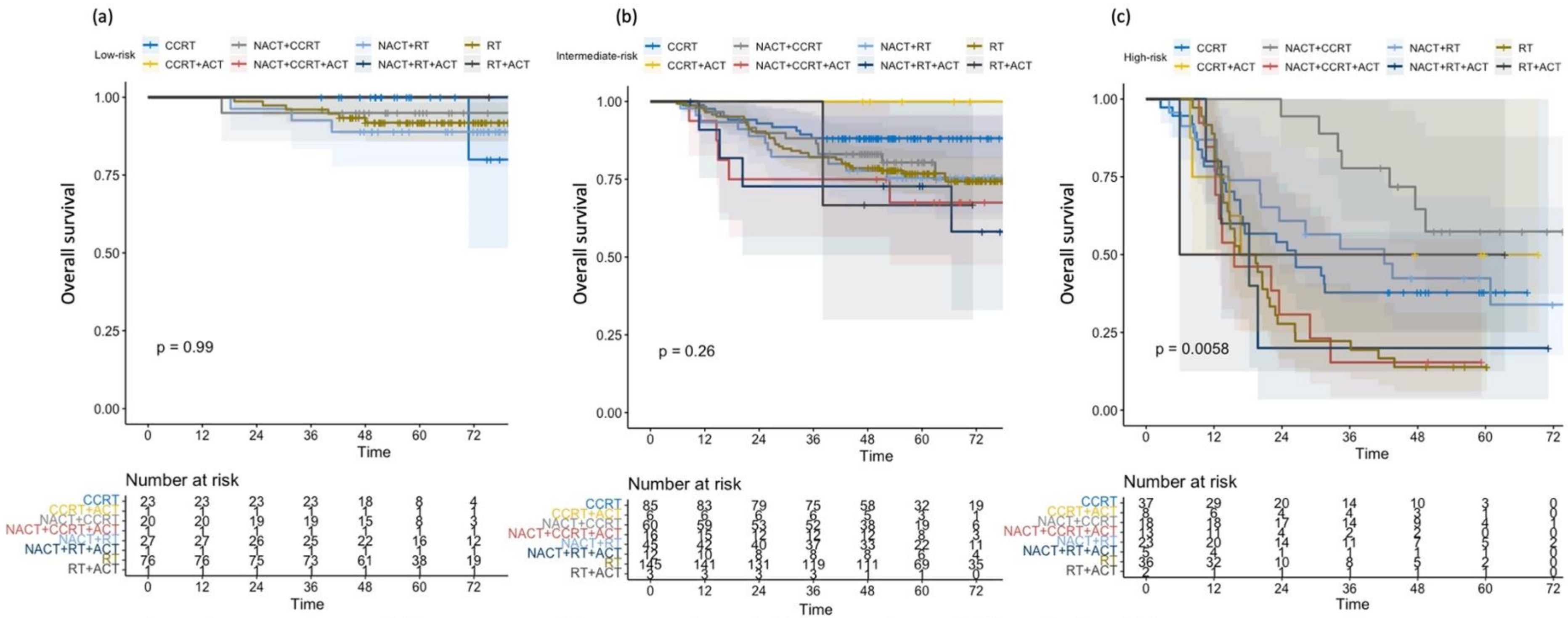

Figure S1: The Kaplan-Meier curves of all treatment modalities in RPA low-risk (a), intermediate-risk (b), and high-risk (c) group.

Abbreviations: RPA: recursive partitioning analysis, RT: Radiotherapy, NACT: Neoadjuvant therapy, ACT: adjuvant therapy, CCRT: Concurrent chemoradiotherapy
